# Supplementary material for: Understanding the mechanism of red light-induced melatonin biosynthesis facilitates the engineering of melatonin-enriched tomatoes
Source: Nat Commun. 2023 Sep 8;14:5525. doi: 10.1038/s41467-023-41307-5 (PMC10491657; doi:10.1038/s41467-023-41307-5)
Supplement: Supplementary file 1 — Supplementary Information [file 41467_2023_41307_MOESM1_ESM.pdf]

Understanding the mechanism of red light-induced melatonin biosynthesis facilitates the engineering of melatonin-enriched tomatoes

Zixin Zhang and Yang Zhang *et al.*

Supplementary Figures

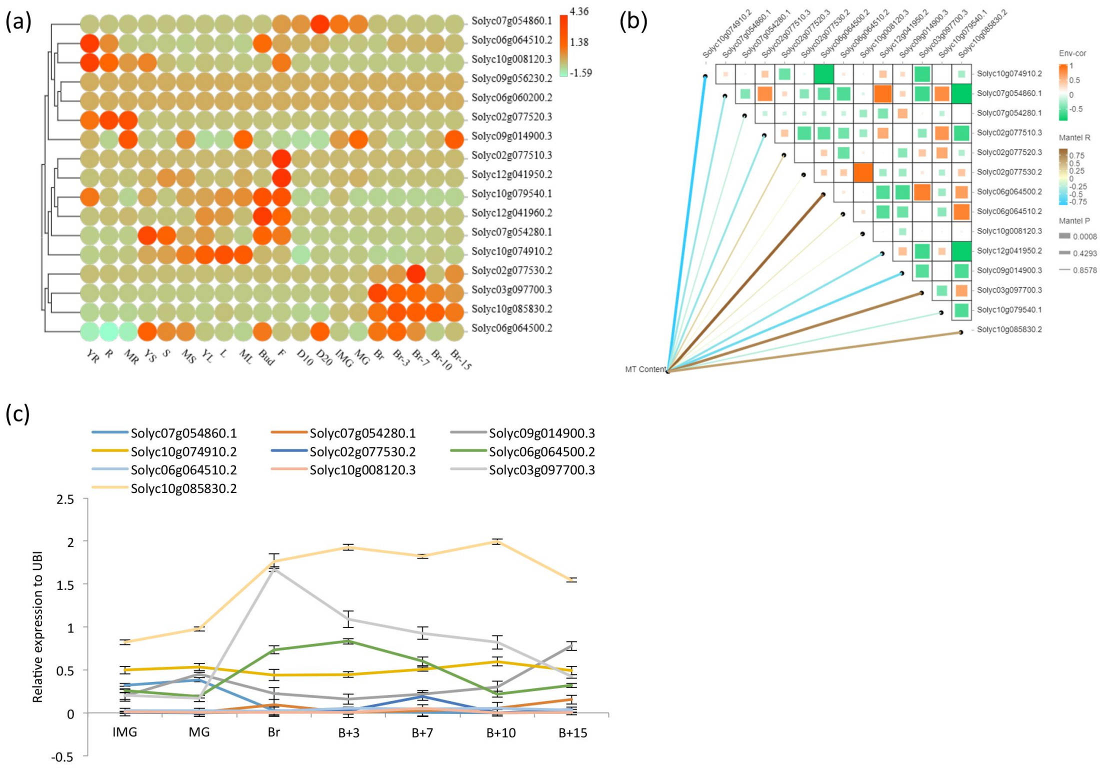

Fig. S1 Screening of key structural genes for melatonin synthesis.

(a) The expression analysis heat map of melatonin synthase gene obtained by screening from MMN Database. (b) Correlation analysis between melatonin content and structural genes. (c) Identification of melatonin synthase gene by RT-qPCR. Data are represented as Mean ± SD (n=3). One biological replicate is the pool of 10-12 fruit from the same seedling. (associated with Fig. 1)

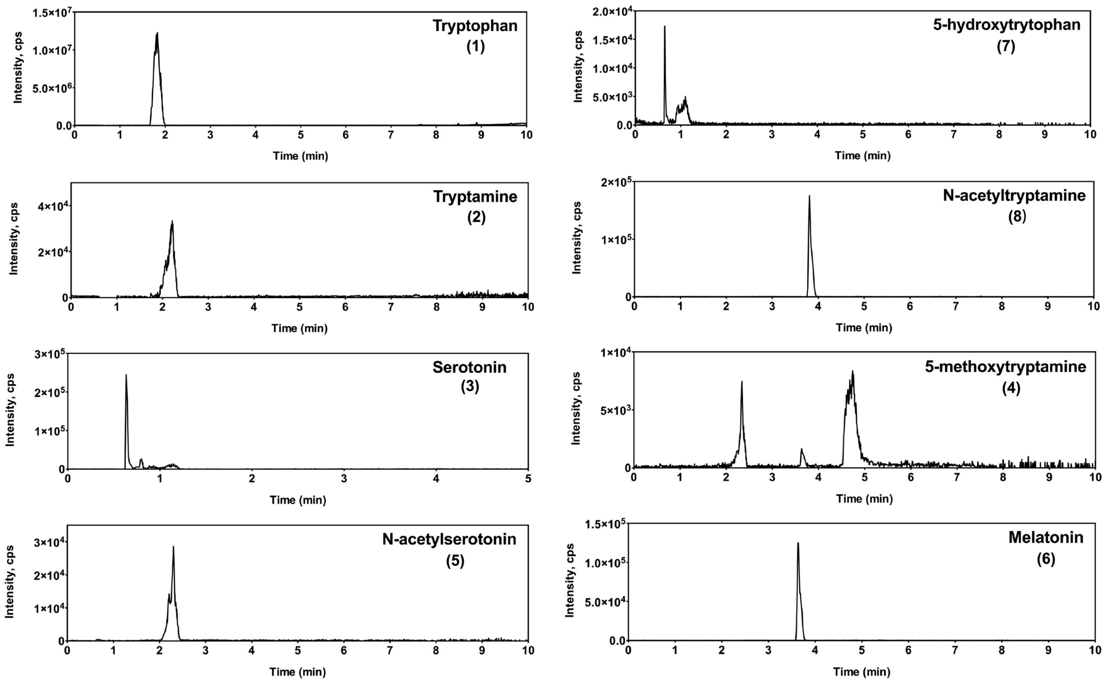

Fig. S2 Peak time of melatonin and its intermediates detected by LC/MS.

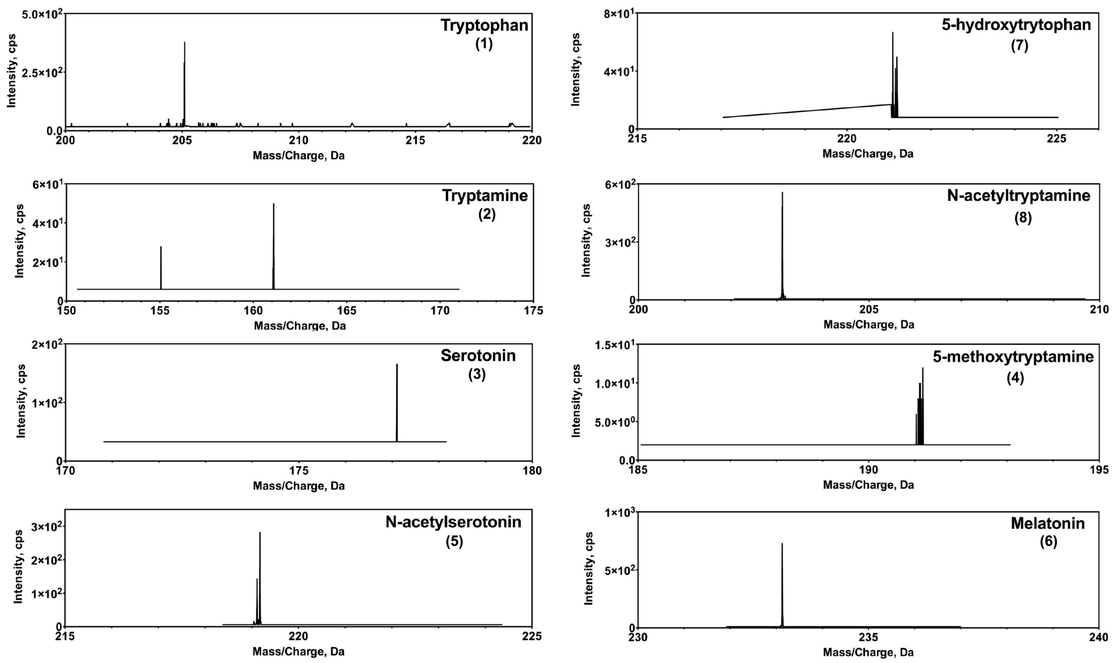

Fig. S3 Molecular weight mass spectra of melatonin and its intermediates detected by LC/MS.

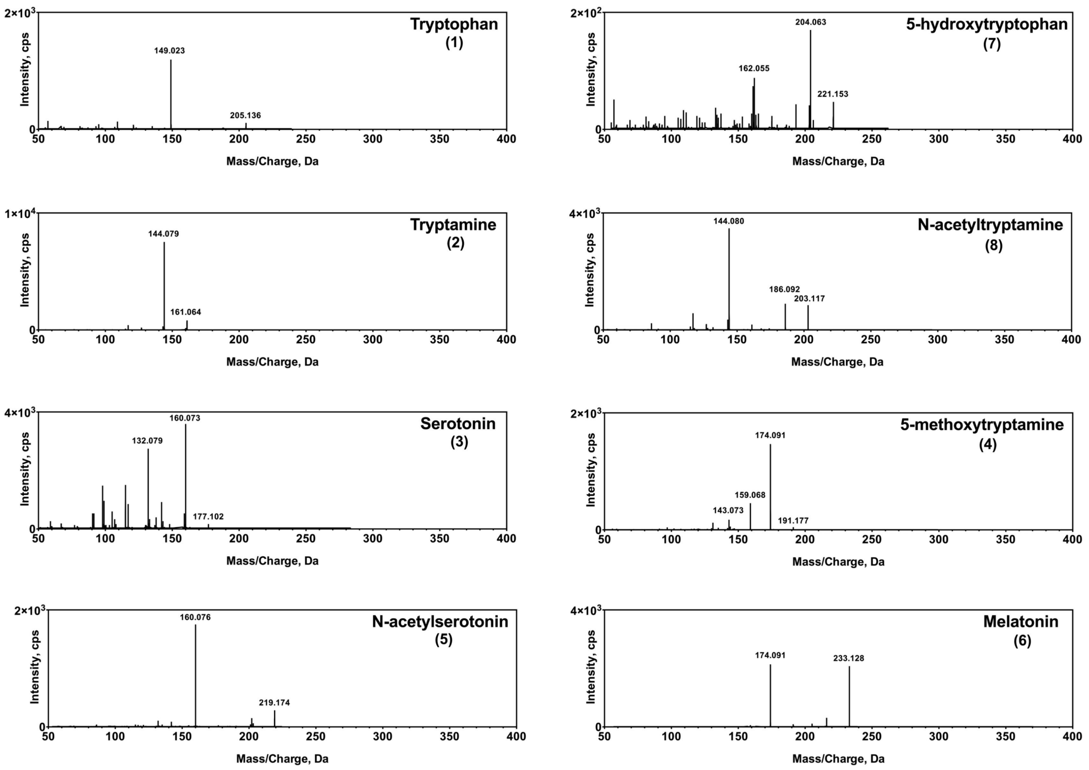

Fig. S4 The MSII spectrum of melatonin and its intermediates detected by LC/MS.

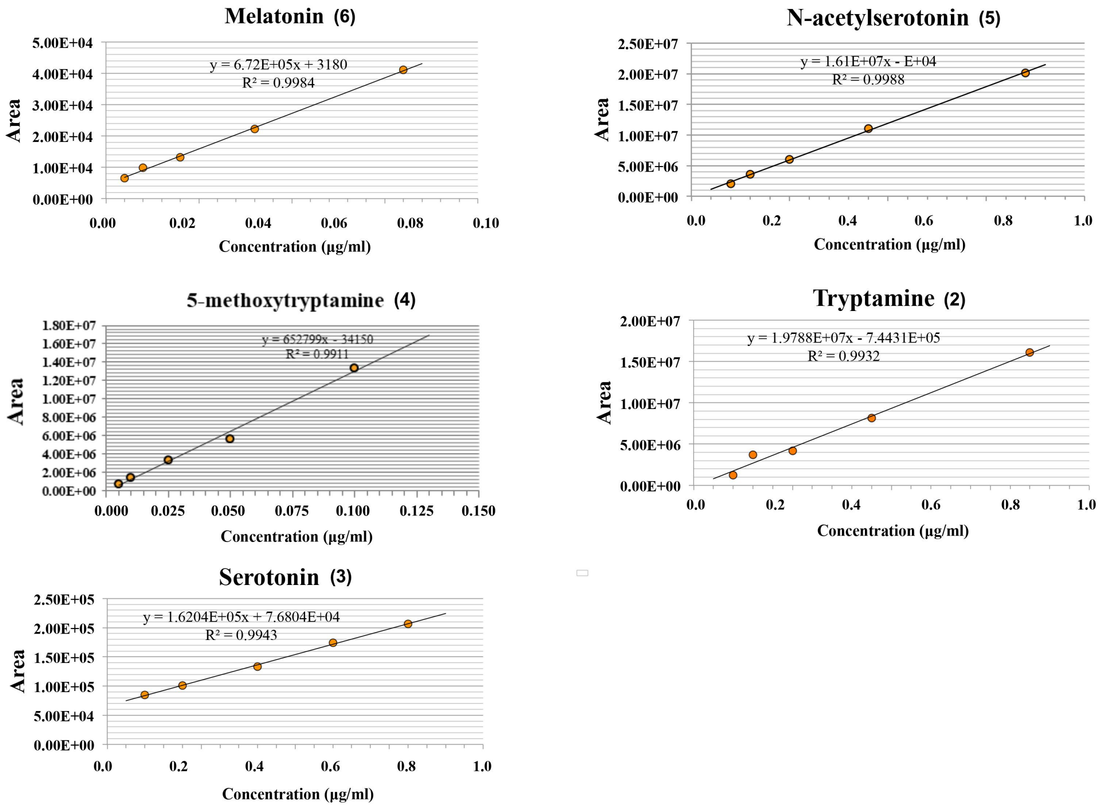

Fig. S5 Standard curve of melatonin and its intermediates.

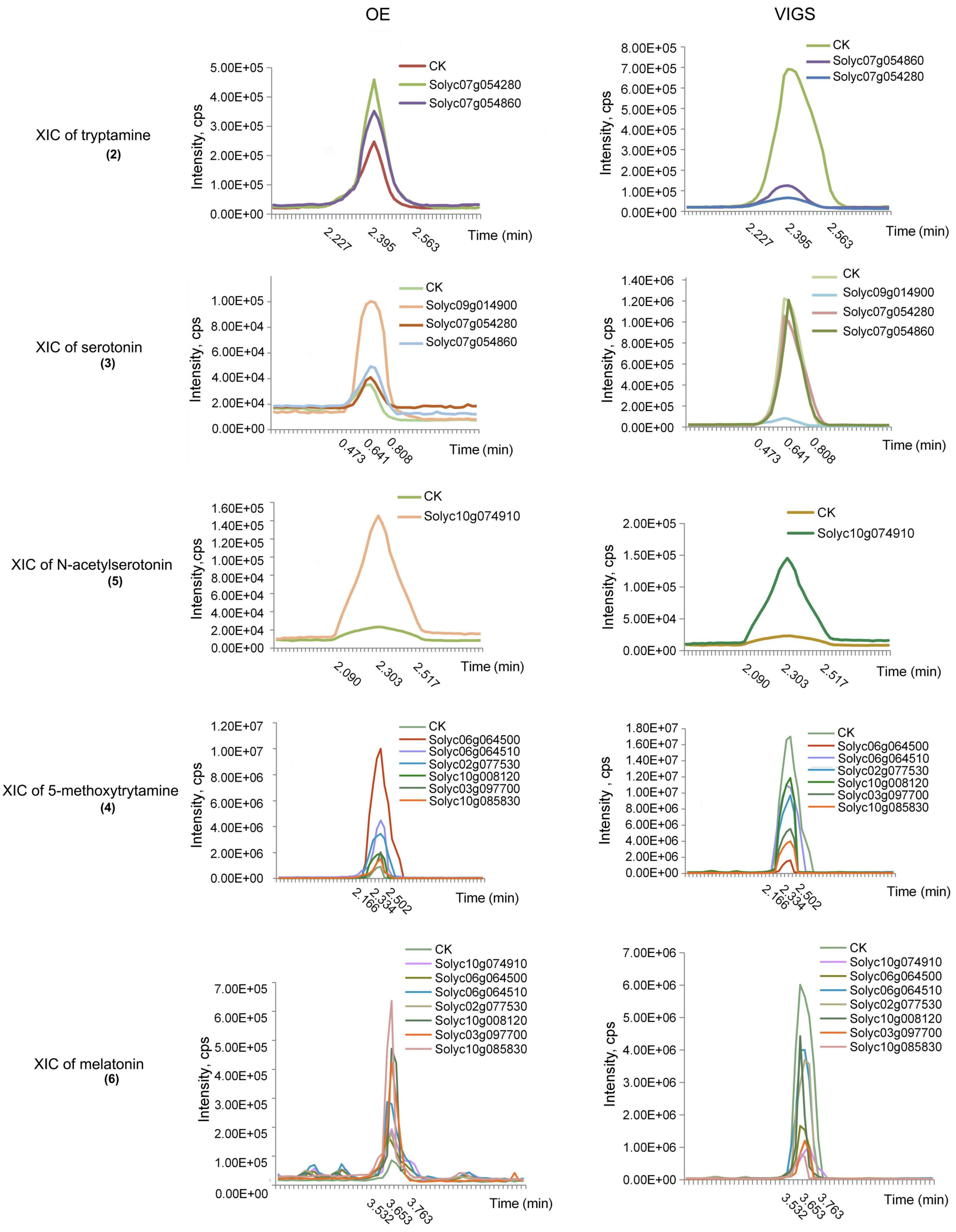

Fig. S6 The extracted ion chromatogram (XIC) of melatonin and its intermediates detected by LC/MS in instantaneously transformed samples. (associated with Fig. 1c).

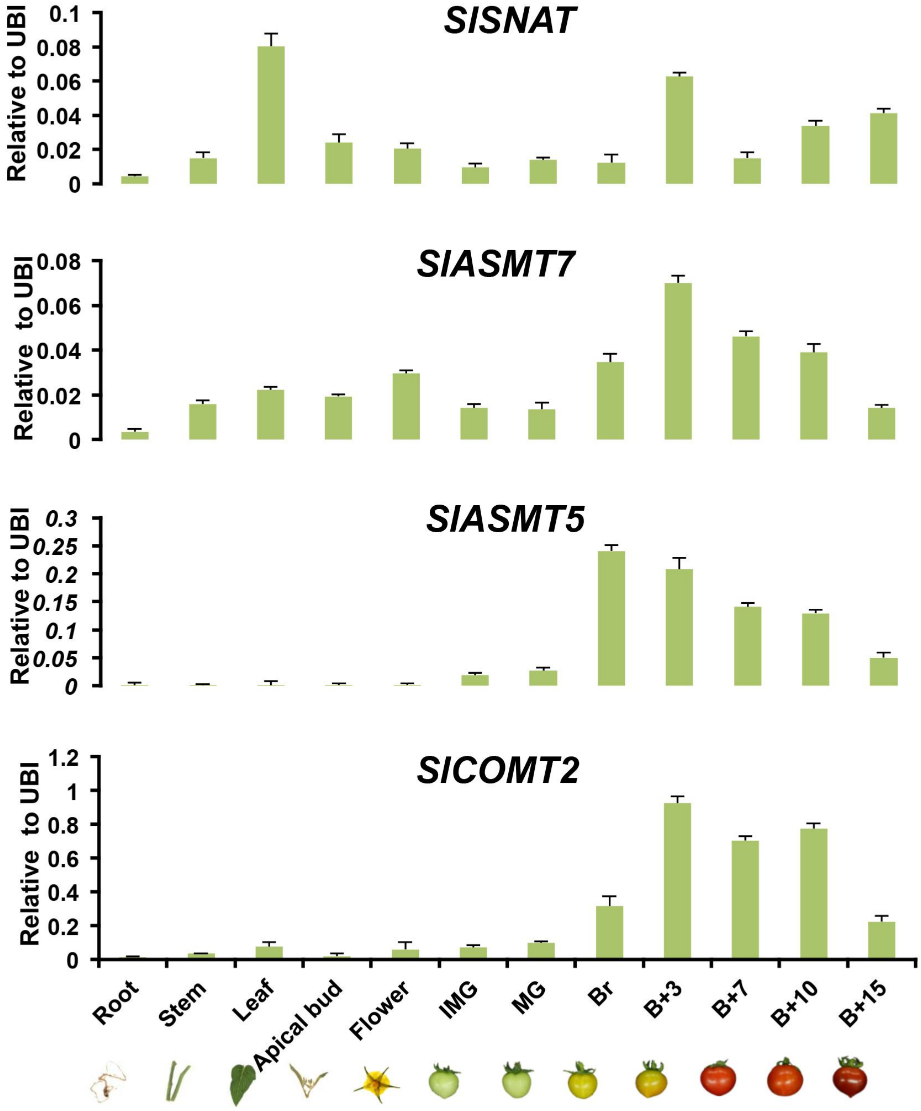

Fig. S7 Tissue expression quantification of four melatonin biosynthetic genes (*SlSNAT*, *SlASMT7*, *SlASMT5*, *SlCOMT2*). Data are represented as Mean ± SD (n=3). In which 10-12 individual issues were pooled as one biological replicate.

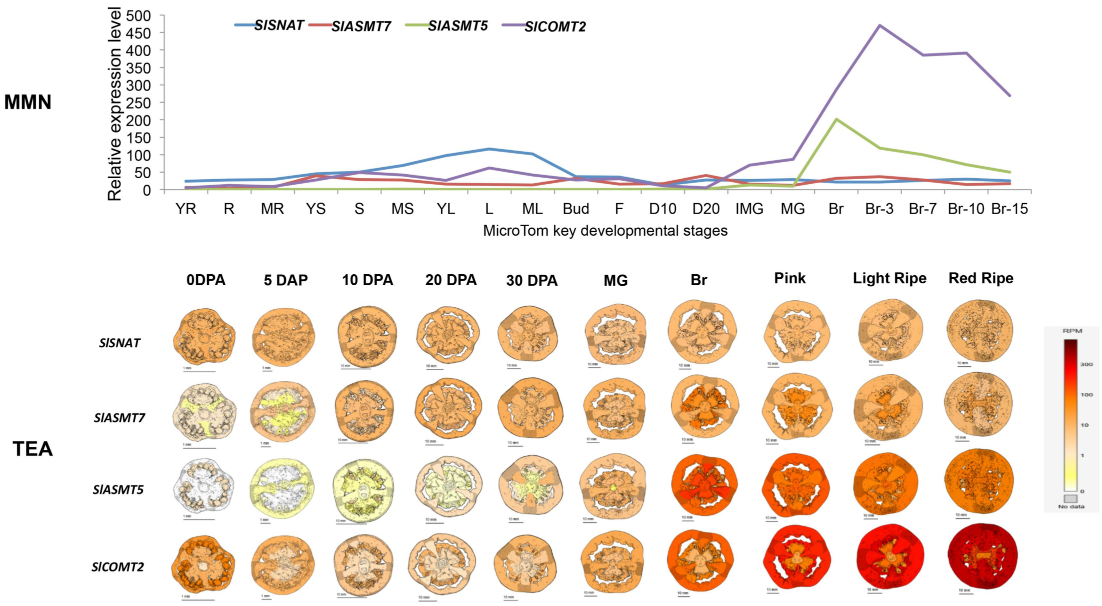

Fig. S8 The expression profile of four melatonin biosynthetic genes (*SlSNAT*, *SlASMT7*, *SlASMT5*, *SlCOMT2*) in MMN [34] and TEA databases [35].

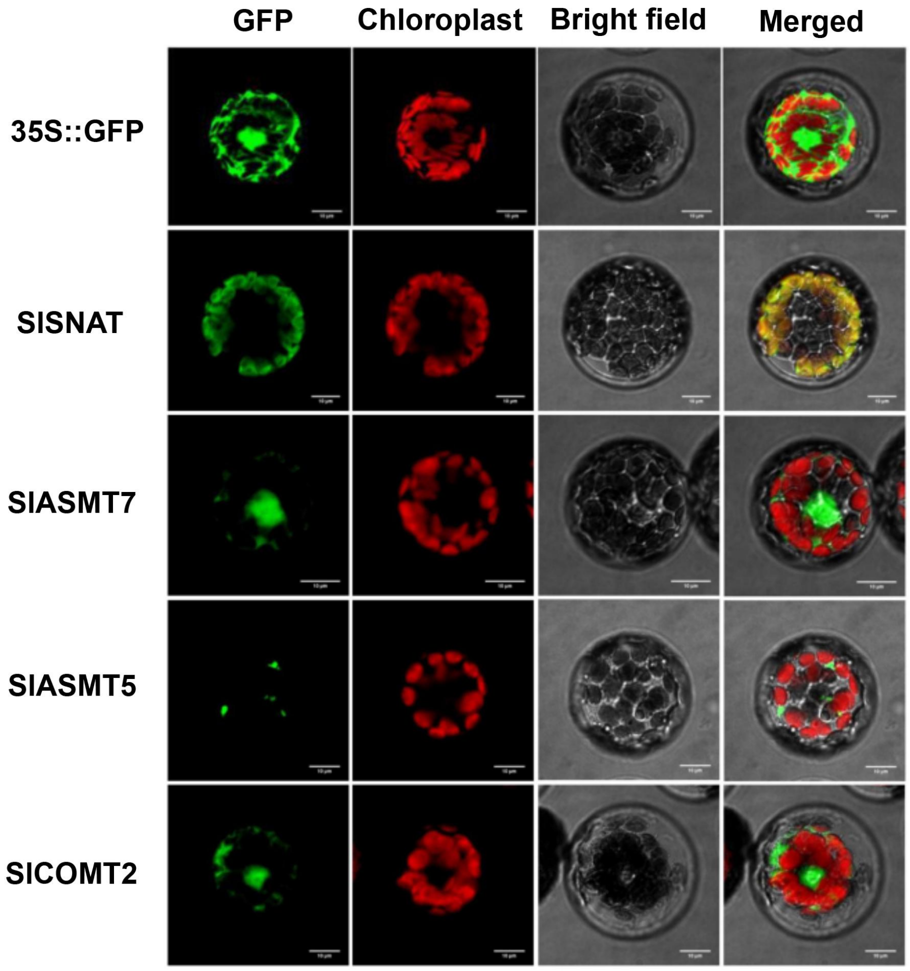

Fig. S9 The proteins' subcellular localization in *Arabidopsis* protoplasts.

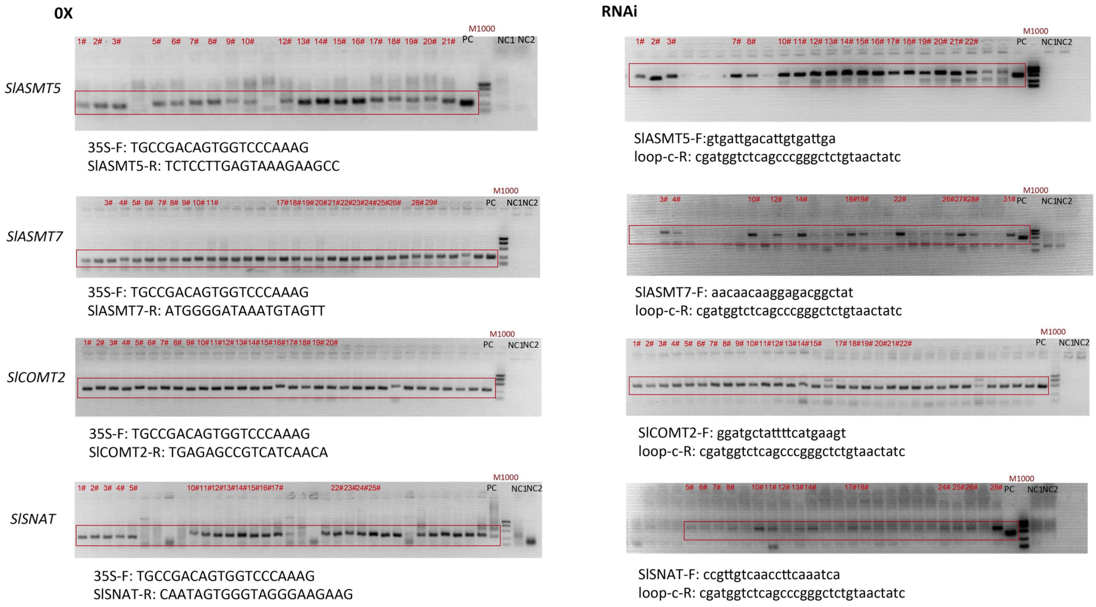

Fig. S10 Genotyping of the overexpressing and RNAi interfering T_0 lines for four melatonin biosynthetic genes (*SlSNAT*, *SlASMT7*, *SlASMT5*, *SlCOMT2*). PC represents the positive control, which was performed by the construction plasmid, and NC1 and NC2 refers to negative control, NC1 was a template from WT, NC2 was a template from the ddH$_2$O. The respective detection primers are at the bottom of each map. (associated with Fig. 2a). Source data are provided as a Source Data file.

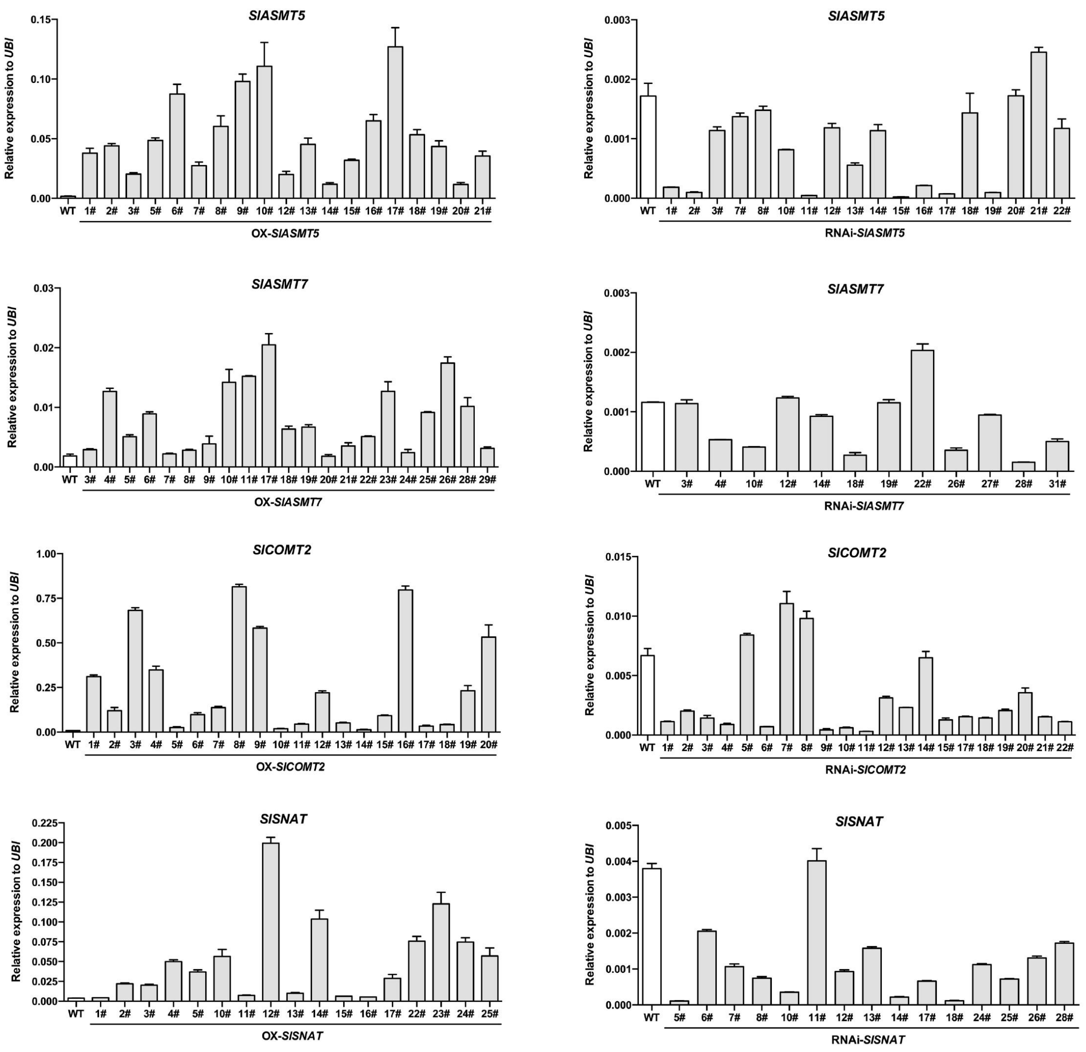

Fig. S11 Transcript level of four melatonin biosynthetic genes (*SlSNAT*, *SlASMT7*, *SlASMT5*, *SlCOMT2*) in the overexpressing and RNAi interfering T_0 lines. Data represent the mean ± SD (n=3). The expression level of one T_0 fruit at Br+3 was calculated as one biological replicate. (associated with Fig. 2a).

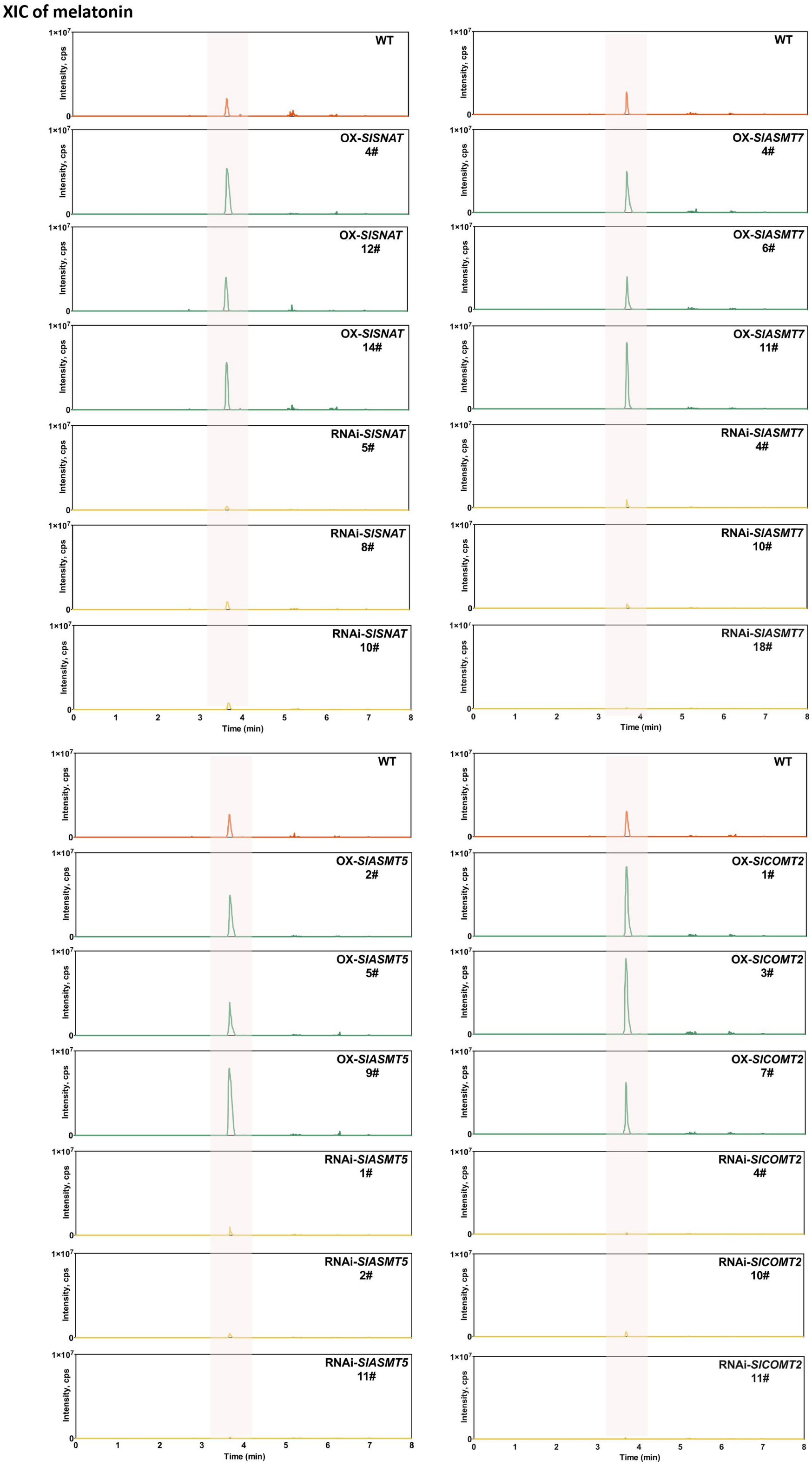

Fig. S12 The extracted ion chromatogram (XIC) of melatonin detected by LC/MS in stable transgenic tomato. (associated with Fig. 2a). Source data are provided as a Source Data file.

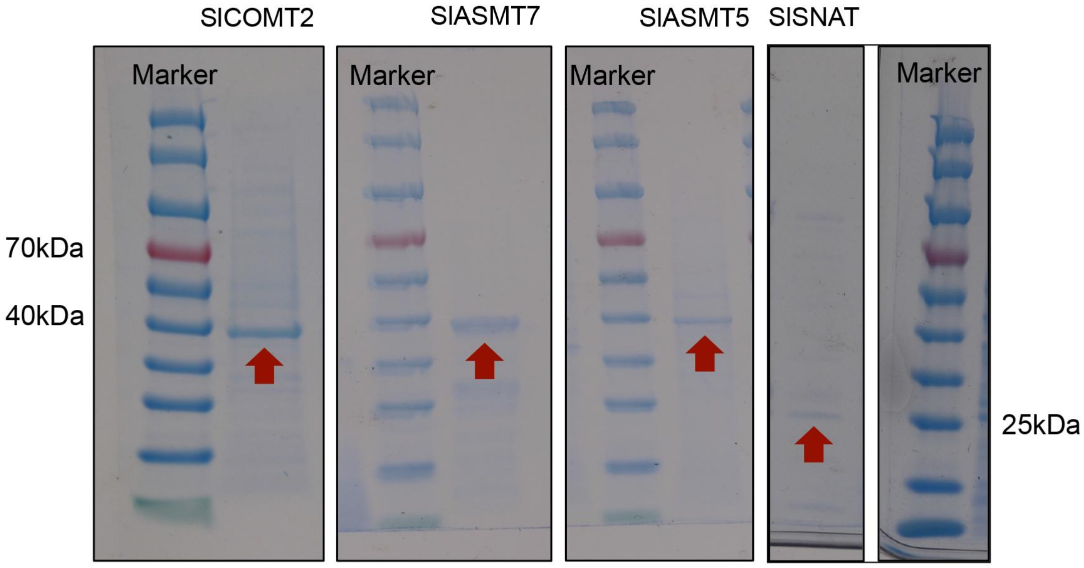

Fig. S13 The purification of SlCOMT2, SlASMT7, SlASMT5 and SlSNAT. (associated with Fig. 2b). Source data are provided as a Source Data file.

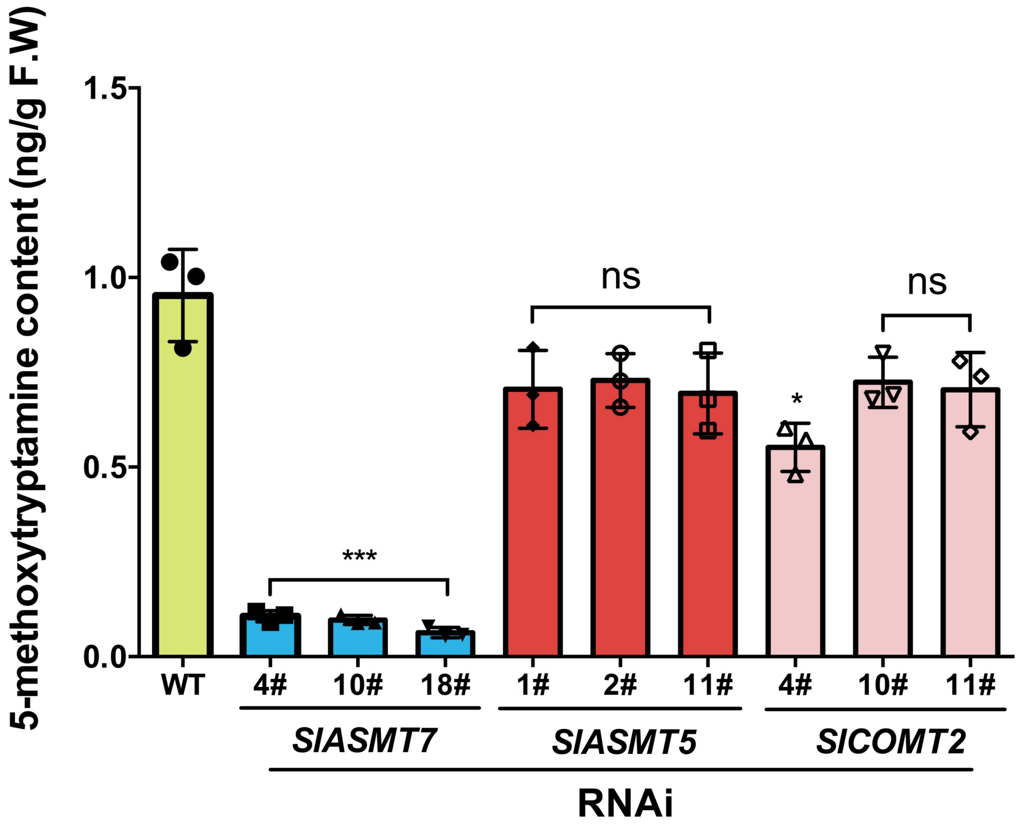

Fig. S14 Content of 5-methoxytrytamine (4) in the fruit of WT and T1 transgenic lines. 5-methoxytrytamine **(4)** content of WT and T1 RNAi fruits at the Br+3 stage. Data is represented as Mean ± SEM (n=3). 10-12 tomato fruits from the same seedling were pooled as one biological replicate. (***P=0.0003, *SlASMT7* 4#), (***P=0.0003, *SlASMT7* 10#), (***P=0.0002, *SlASMT7* 18#), (*P=0.0072, *SlCOMT2* 4#), ns indicates not significant (P>0.05). * indicates significant difference from WT analyzed by two-sided Student’ s t test.(associated with Fig. 3b).

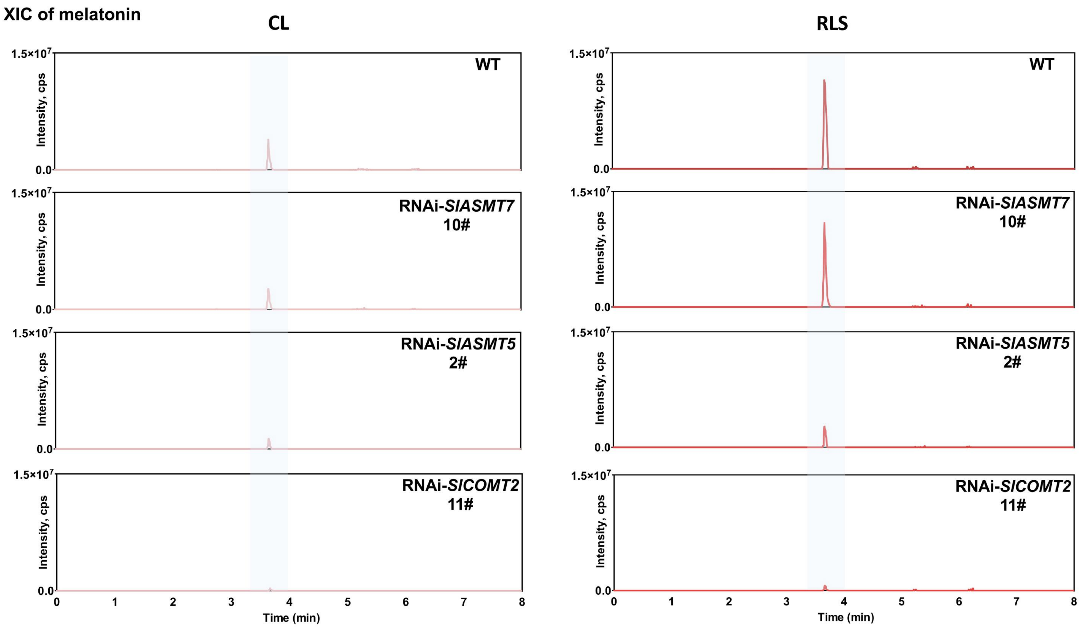

Fig. S15 The extracted ion chromatogram (XIC) of melatonin detected by LC/MS in wild-type and transgenic tomato fruit under control light [1] and red light supplement (RLS). (associated with Fig. 3c). Source data are provided as a Source Data file.

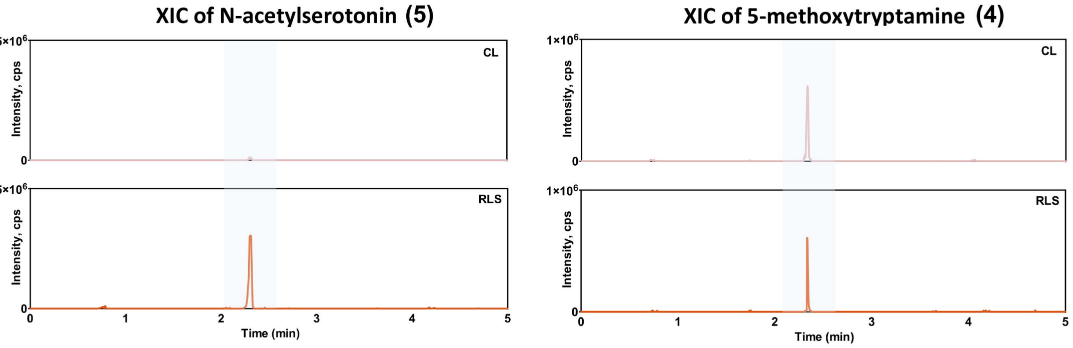

Fig. S16 The extracted ion chromatogram (XIC) of N-acetylserotonin (5) and 5-methoxytryptamine (4) detected by LC/MS in wild-type tomato fruit under control light [1] and red light supplement (RLS). (associated with Fig. 3b). Source data are provided as a Source Data file.

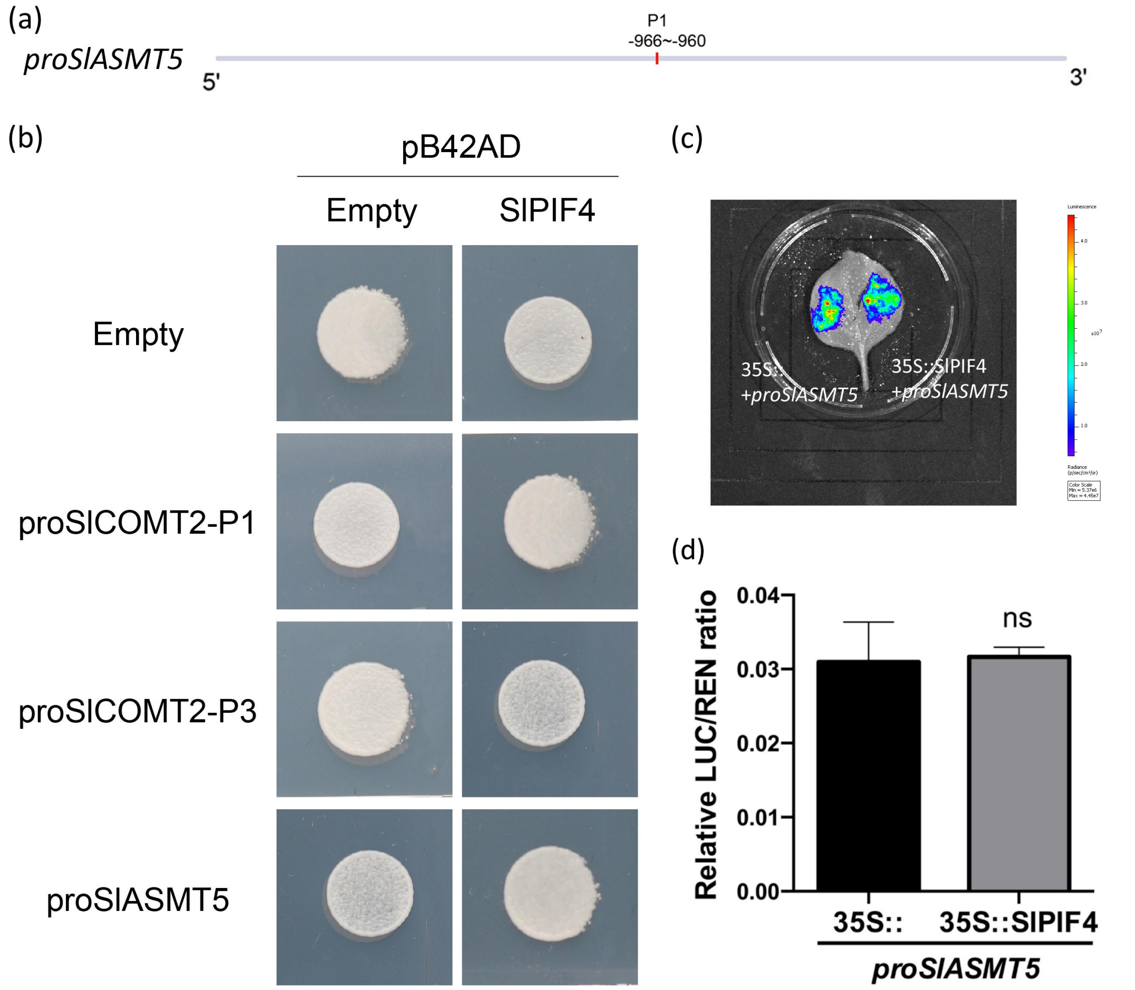

Fig. S17 Interactions of SlPIF4 proteins and the promoters of *SlCOMT2*. (a) A schematic of the promoter of SlASMT5. (b) Interactions of SlPIF4 proteins and the G-BOX fragment of proSlCOMT2 (P1, P3) and the 2000bp sequence of proSlASMT5 in yeast cells. (c) Interactions of SlPIF4 protein and the *proSlASMT5* confirmed with dual luciferase reporter assays in *Nicotianabenthamiana* leaves. 35S::+*proSlASMT5* were used as controls. (d)Transient expression analysis in protoplasts of tomato. LUC/REN is the average ratio of the bioluminescence of firefly luciferase to that of Renilla luciferase. Data is represented as Mean ± SEM (n=3). No significant difference was observed. (associated with Fig. 4).

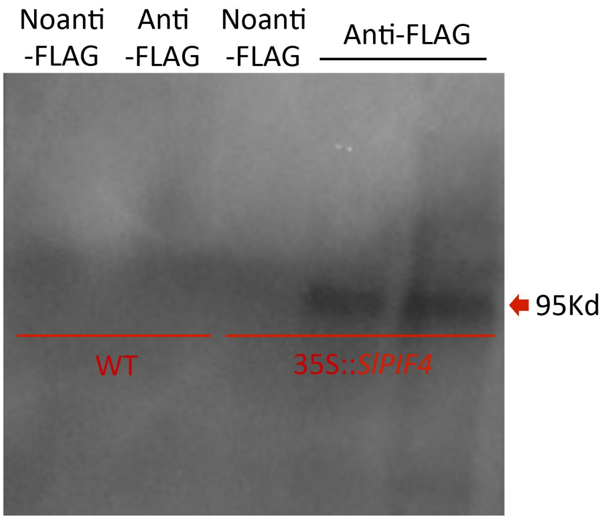

Fig. S18 Western-Blot analysis of the expression of SlPIF4-3×FLAG recombinant protein. Detection of the FLAG-tag in the FLAG fusion expression target protein SlPIF4 in the WT and transgenic lines of 35S::*SlPIF4* for ChIP-qPCR assays. Total protein extracts were immunoprecipitated with an anti-FLAG antibody. The target protein band is 95Kd. (associated with Fig. 4i).

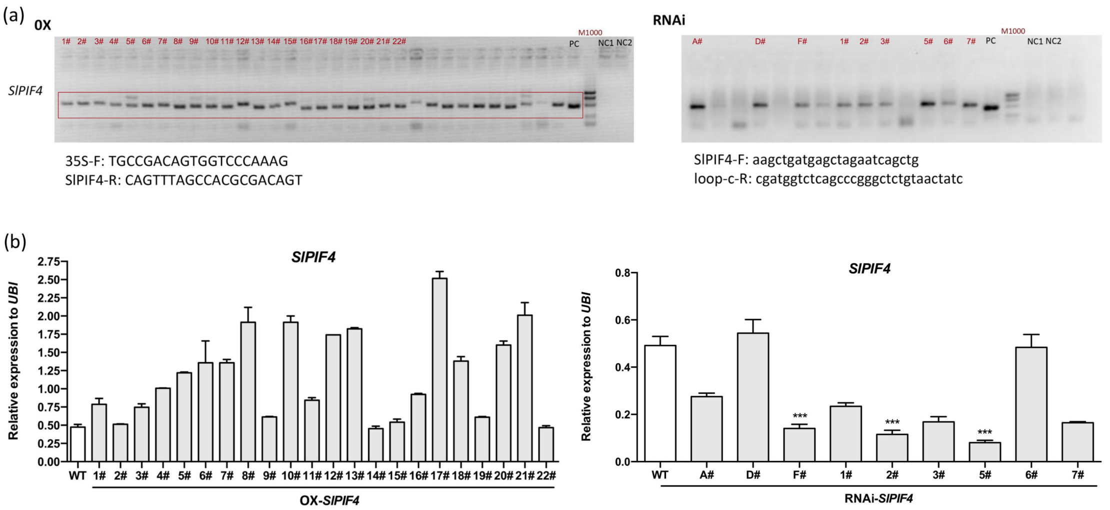

Fig. S19 Identification of *SlPIF4* overexpression and RNAi plants. Genotyping overexpression and RNAi T_0 lines of *SlPIF4*. PC represents the positive control, which was performed by the construction plasmid, and NC1 and NC2 refer to the negative control, NC1 was a template from WT, and NC2 was a template from the ddH_2O. The respective detection primers are at the bottom of each map. (b) The transcript level of *SlPIF4* in T_0 overexpression and RNAi lines. The expression level of one tomato fruit at Br+3 was calculated as one biological replicate. Data represent the mean ± SD (n=3). ***P < 0.001, (Student's *t* test). (associated with Fig. 3b). Source data are provided as a Source Data file.

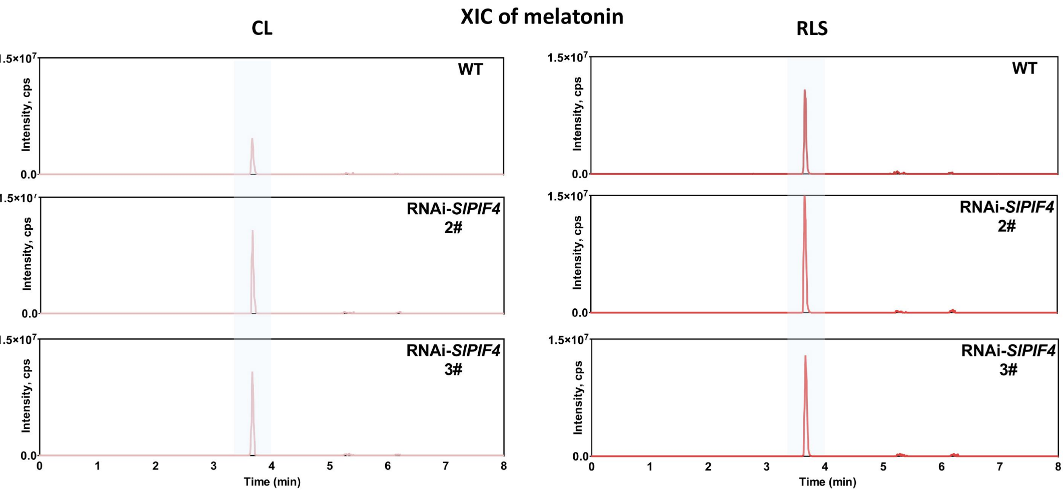

Fig. S20 The extracted ion chromatogram (XIC) of melatonin detected by LC/MS in wild-type and RNAi-*SlPIF4* transgenic tomato fruit under control light [1] and red light supplement (RLS). (associated with Fig. 4i). Source data are provided as a Source Data file.

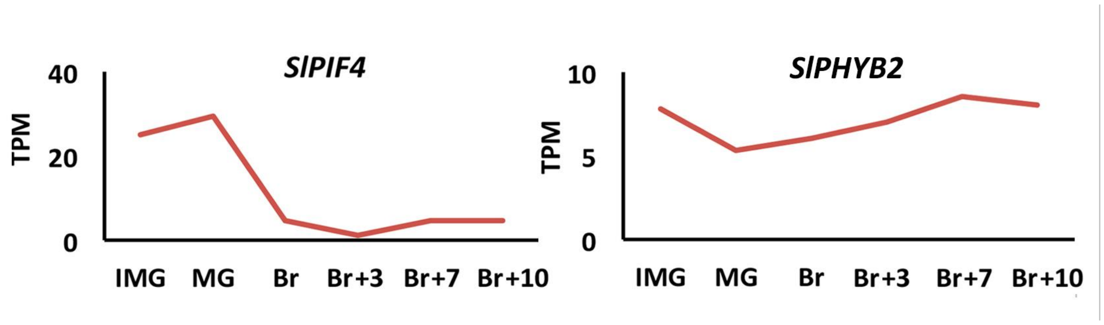

Fig. S21 The expression trend of *SlPHYB2* and *SlPIF4* in different fruit development stages in the MMN database [34] is the opposite. (associated with Fig. 5).

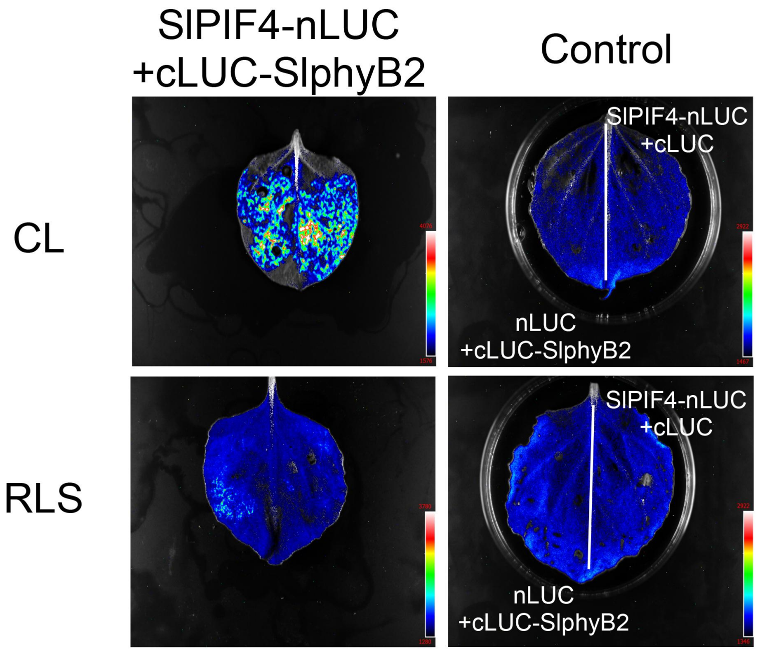

Fig. S22 Quantitative analysis of luminescence intensity showing the interaction between SlphyB2 and SlPIF4 in *Nicotiana benthamiana* leaves. (associated with Fig. 5b).

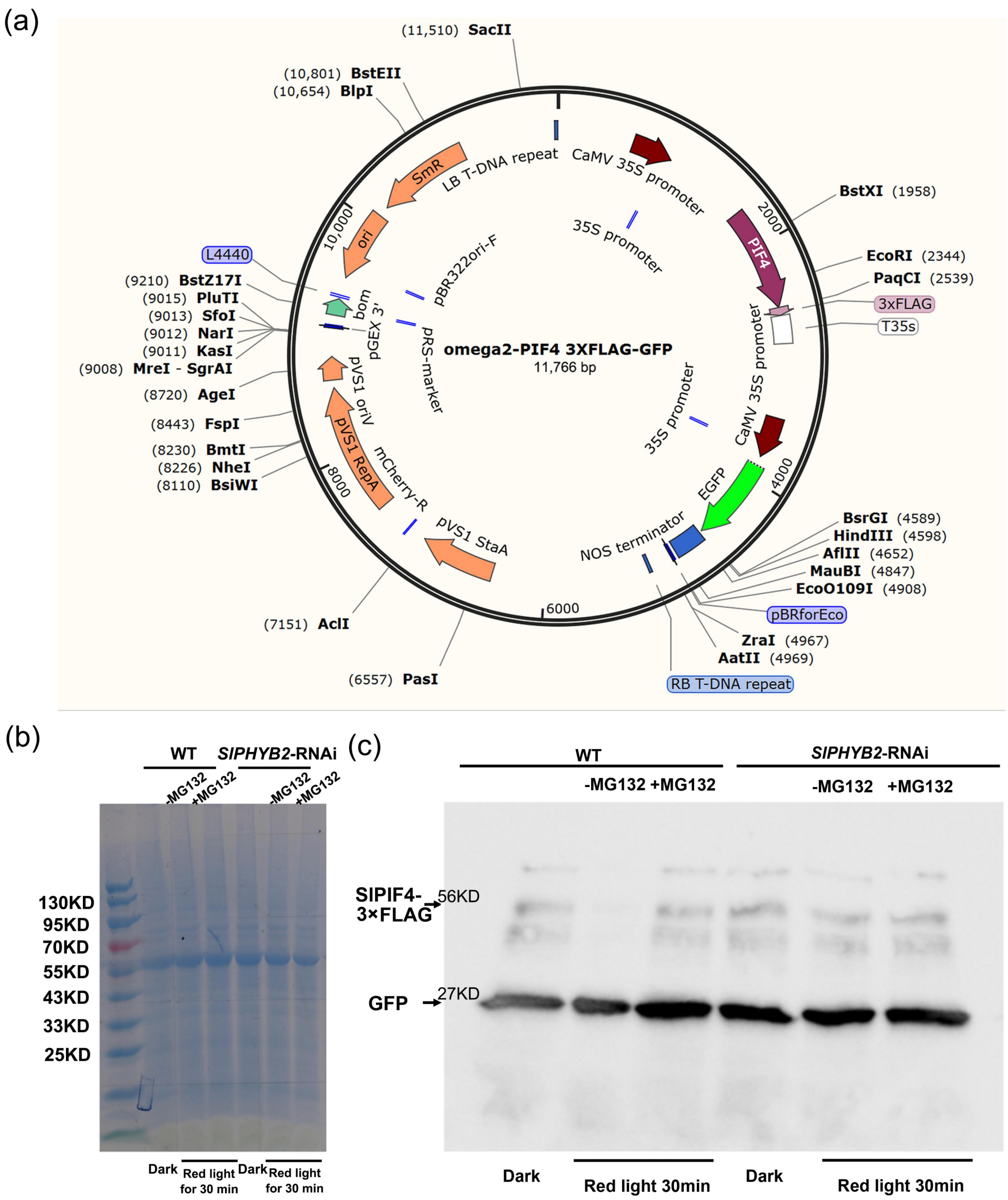

Fig. S23 Red light response of the degradation of SlPIF4 by SlphyB2. (a) Constructed plasmid map. (b) SDS-PAGE detects the total protein loading. (c) Western blot detection of ubiquitination degradation of SlPIF4 mediated by SlphyB2. (associated with Fig. 5d). Source data are provided as a Source Data file.

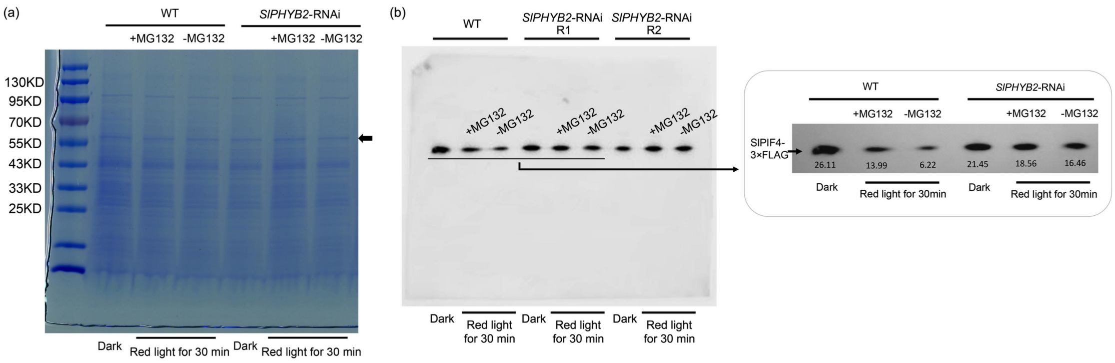

Fig. S24 The detection by western blot of total protein in WT and transgenic plants of RNAi-*SlPHYB2*. (a) SDS-PAGE detects the total protein loading. The black arrow marks the position of the SlPIF4 protein as calculated according to the size of the protein. (b) Full immunoblot image of western-Blot detecting for degradation of SlPIF4 protein. R1 and R2 are two technical replicates. (associated with Fig. 5b). Source data are provided as a Source Data file.

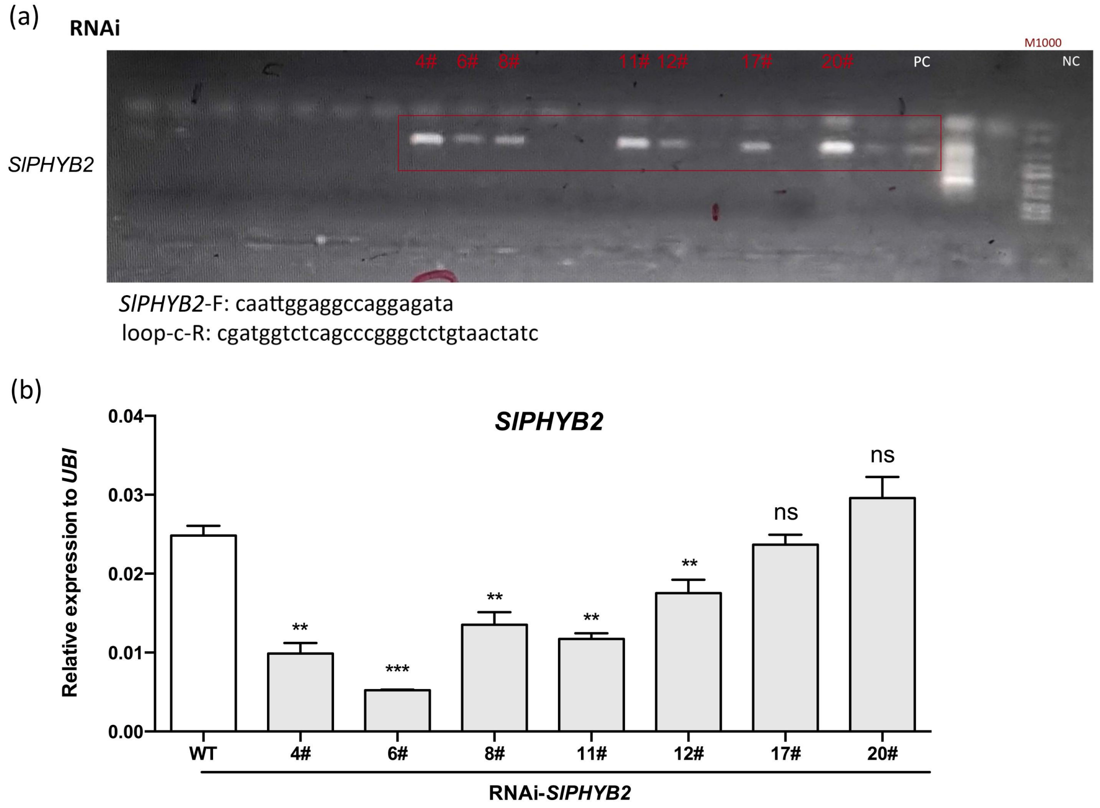

Fig. S25 Identification of *SlPHYB2* RNAi lines.

(a) Genotyping T_0 *SlPHYB2* RNAi lines. NC refers to the negative control, NC was a template from WT. The detection primers are at the bottom of the gel. (b) The transcript level of *SlPHYB2* in T_0 RNAi lines. The expression level of one tomato fruit at Br+3 was calculated as one biological replicate. Data represent the mean ± SD (n=3). **P < 0.01, (Student's *t* test). ns indicates not significant (P>0.05). (associated with Fig. 5e, f). Source data are provided as a Source Data file.

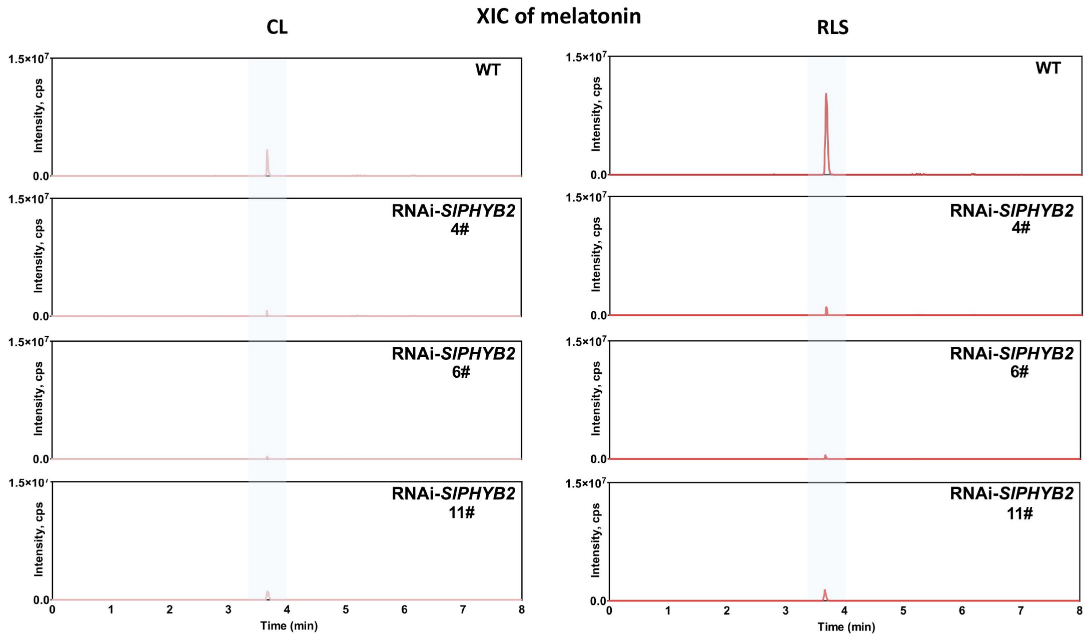

Fig. S26 The extracted ion chromatogram (XIC) of melatonin detected by LC/MS in wild-type and RNAi-*SlPHYB2* transgenic tomato fruit under control light [1] and red light supplement (RLS). (associated with Fig. 5f). Source data are provided as a Source Data file.

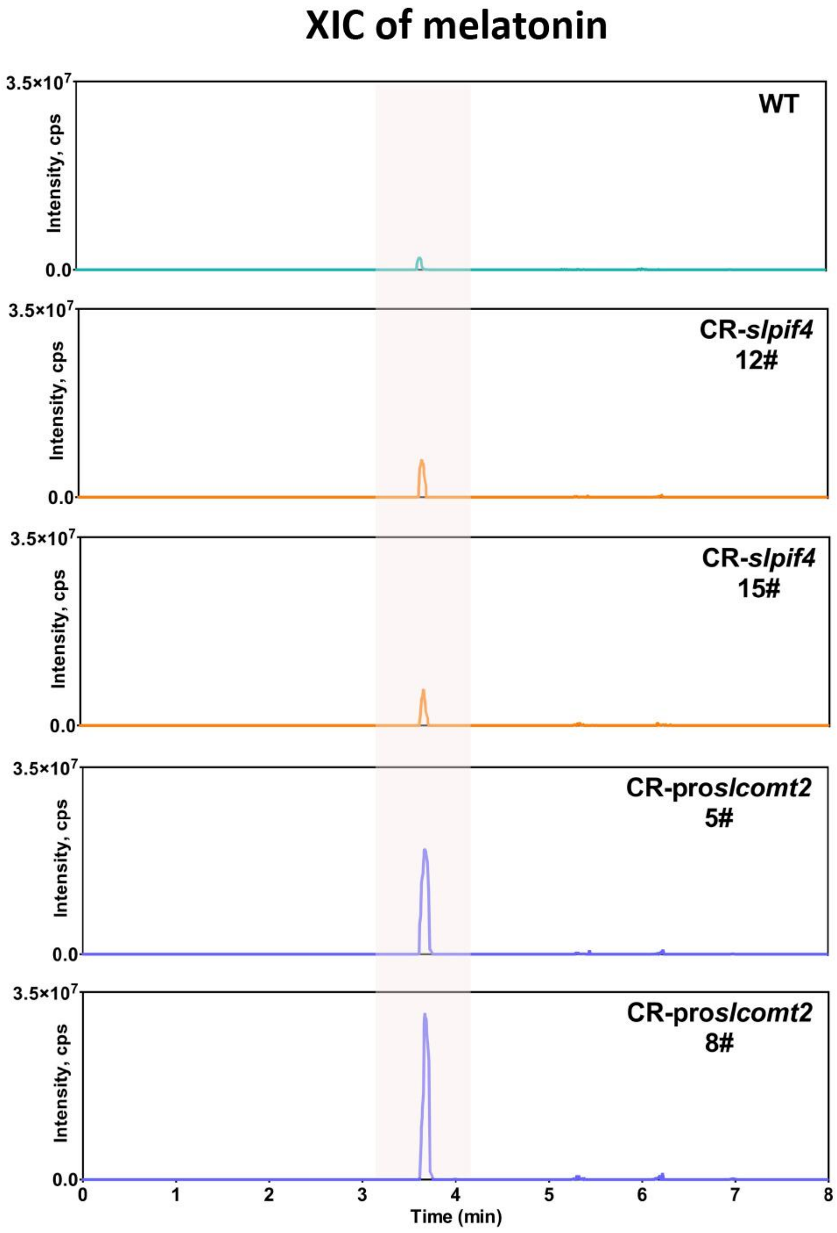

Fig. S27 The extracted ion chromatogram (XIC) of melatonin detected by LC/MS in wild-type and T2 CRISPR (CR) tomato fruit. (associated with Fig. 7). Source data are provided as a Source Data file.

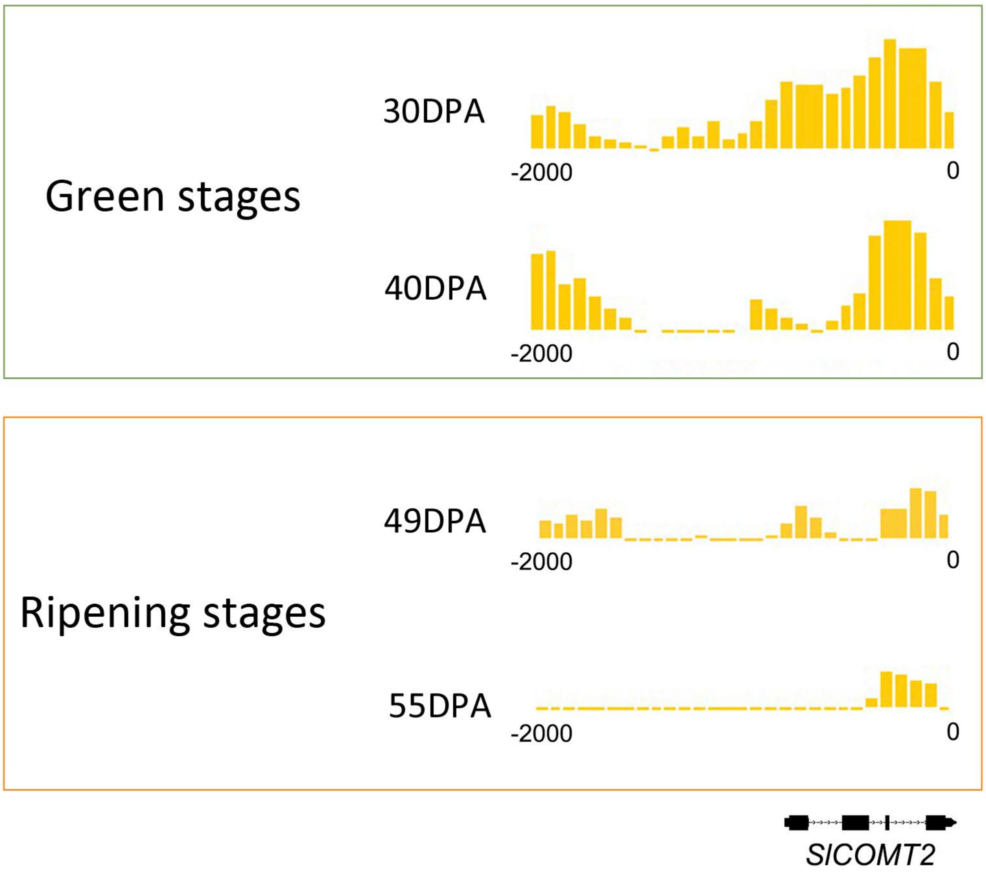

Fig. S28 The DNA methylation rate of *SlCOMT2* at the green stages (30DPA, 40DPA) and ripening stages (49DPA, 55DPA). DPA (days after anthesis).

Reference

1. Clough, S.J.A.B., A.F. Floral Dip: A Simplified Method for Agrobacterium-Mediated Transformation of *Arabidopsis thaliana*. *Plant J.* **16**, 735-743 (1998).

Supplementary Tables

Table S1. The primers used for gene cloning

Gene	Primers' name	Sequence (5'-3')
SlT5H	SlT5H-F	ATGGAAGCATCAATTCTACAGCTAC
	SlT5H-R	TTACAACTTGTTGATGGTTGGAACAAG
SlTDC280	SlTDC280-F	ATGGGAACCCTTAATTCAAATAACAAC
	SlTDC280-R	CTAAAACACAATTTTTCTGAGCAAATCAT
SlTDC860	SlTDC860-F	ATGGGAAGCCTTGATTCCAA
	SlTDC860-R	TTAAAACACACTTTTTCTCAGCAAACC
SlSNAT	SlSNAT-F	ATGCAAACTCTCCACTTAGTATCCAC
	SlSNAT-R	CTAATACATGGGGTACCAGAACATTCC
SlAMST7	SlAMST7-F	ATGGCCAGTAACAACAATATTTGT
	SlAMST7-R	TCTCTTTTGAAGCATAAGTAATCCTCA
SlAMST5	SlAMST5-F	ATGGCATTGCCTAATAATATTGGAGAC
	SlAMST5-R	ATCAAGGGTAAACCTCAATAACAGACC
SlCOMT2	SlCOMT2-F	ATGGAGAATGCAAATGGGGAAC
	SlCOMT2-R	CATGACTTGTGAAATTCAAGAATC
SlphyB2	SlphyB2-F	ATGGCTTCTGGGAGTGGA
	SlphyB2-R	TTGAATCAGCATTGGTAGTTGA

		AGG
SlPIF4	SlPIF4-F	ATGGGATTTGATCATGAGC
	SlPIF4-R	AGTGGCAGGTGCATTACTA
Solyc10g008120.3	8120-F	ATGGTTGTGCCAATTGTTGG
	8120-R	TTAAGGATAAACCTCAATTAGTGACCTTAATCC
Solyc02g077530.3	530-F	ATGGAAACAAATAATAATGTTGAGAGAGC
	530-R	TTAAGGGAAAACTTGAATGAGGGAC
Solyc06g064510.2	510-F	ATGGCCAATAAGAAGAACAACAT
	510-R	TTTAAGGATAAACTTCCATTAGAGACCT

Table S2. The primers used for qRT-PCR

Gene	Primer	Sequence (5'-3')
SlT5H	SlT5H-RT-F	TCGGCCAAATTCCGACTCTA
	SlT5H-RT-R	GAGCAACCGAAGGAGAGGTA
SlTDC280	SlTDC280-RT-F	TTTCACTCGTGTGCTTTCGG
	SlTDC280-RT-R	TATCCTCAGTGAACGTCGCA
SlTDC860	SlTDC860-RT-F	TATGGCTCGTCATGCGTAGT
	SlTDC860-RT-R	GGCACGACAACTTCAAACCT
SlSNAT	SlSNAT-RT-F	TCAGGGACAAGGACTTGGAA
	SlSNAT-RT-R	TTCCCTCTGGATCAGGTTCA
SlAMST7	SlAMST7-RT-F	GACGTCATTGGTGGACATCG
	SlAMST7-RT-R	TGCGTTGGCATGAGGAATTT
SlAMST5	SlAMST5-RT-F	CGAAGCTATGGGATGTGACG
	SlAMST5-RT-R	CCTTCCAAGCCTTCAACCAC
SlCOMT2	SlCOMT2-RT-F	TGTCTGGAGTTTCTGTGCCA
	SlCOMT2-RT-R	CAACCACCTCAGGCAAATCA
SlphyB2	SlphyB2-RT-F	TGCCGTTGATGTTGAAGGTC
	SlphyB2-RT-R	GCGCAGTTGTCTGTGATTCT
SlPIF4	SlPIF4-RT-F	TGCAACTGCAGATGATGTGG
	SlPIF4-RT-R	GTAGTGTTGGACACCAGGGA
Solyc10g008120.3	8120-RT-F	TGCCTCATGTAGTTGAAGGACT
	8120-RT-R	TTCCTTGCTGGGTATCGCTT
Solyc02g077530.3	530-RT-F	GGCACTGGCGTTATAGCTAAGAC
	530-RT-R	GAGGGACCTTAGTCCAAGGAGAGG
Solyc06g064510.2	510-RT-F	TCTCTGACCTCATTGCTGCTTTGC
	510-RT-R	CATTGCTACGGTGCCGGTTCC

Table S3. Primers of the vector construction

Empty vector	Primers' name	Sequence (5'-3')
pGreen Ⅱ 0800-LUC	0800-proSlCOMT2	F: CTCGAGGTCGACGGTATCGATAAGCTTTAATTAATCATGATT[illegible]
		R: GGGCTGCAGGAATTCGATATCAAGCTTGGCTTCTCTATACTT[illegible]
	0800-proSlASMT5	F: CTCGAGGTCGACGGTATCGATAAGCTTTGATTCCTCTGTCTTG[illegible]
		R: GGGCTGCAGGAATTCGATATCAAGCTTTTTTCTTTTGAGATG[illegible]
pCAMBIA1302-GFP	02-SlPIF4	F: GGATCCATGGGATTTGATCATGAGC
		R: GTCGACAGTGGCAGGTGCATTACTA
	02-SlSNAT	F: GGTACCATGCAAACTCTCCACTTAGTATCCAC
		R: GGATCCCTAATACATGGGGTACCAGAACATTCC
	02-SlASMT5	F: GAGCTCATGGCATTGCCTAATAATATTGGAGAC
		R: GTCGACATCAAGGGTAAACCTCAATAACAGACC
	02-SlASMT7	F: GGTACCATGGCCAGTAACAACAATATTTGT
		R: GGATCCTCTCTTTTGAAGCATAAGTAATCCTCA
	02-SlCOMT2	F: GAGCTCATGGAGAATGCAAATGGGGAAC
		R: GTCGACCATGACTTGTGAAATTCAAGAATC
pGEXT4	GST-SlPIF4	F: ATCGGATCTGGTTCCGCGTGGATCCATGGGATTTGATCATGAG[illegible]
		R: GTCGACCCGGGAATTCCGGGGATCCAGTGGCAGGTGCATTACT[illegible]
pB42AD	42AD-SlPIF4	F: CCTTATGATGTGCCAGATTATGCCTCTCCCGAATTCATGGGATT[illegible]
		R: CCAAACCTCTGGCGAAGAAGTCCAAAGCTTCTCGAGAGTGGC[illegible]
pDEST17	His-SlSNAT	F: GGGGACAAGTTTGTACAAAAAAGCAGGCTTAATGCAAACTCT[illegible]
		R: GGGGACCACTTTGTACAAGAAAGCTGGGTACTAATACATGGGG[illegible]
	His-SlASMT7	F: GGGGACAAGTTTGTACAAAAAAGCAGGCTTAATGGCCAGTAA[illegible]
		R: GGGGACCACTTTGTACAAGAAAGCTGGGTATCAAATATTCTTT[illegible]
	His-SlASMT5	F: GGGGACAAGTTTGTACAAAAAAGCAGGCTTAATGGCATTGCC[illegible]
		R: GGGGACCACTTTGTACAAGAAAGCTGGGTATCAAGGGTAAAC[illegible]
	His-SlCOMT2	F: GGGGACAAGTTTGTACAAAAAAGCAGGCTTAATGGAGAATGC[illegible]
		R: GGGGACCACTTTGTACAAGAAAGCTGGGTATCATGACTTGTG[illegible]
pCAMBI	06-SlPIF4	F: GGATCCATGGGATTTGATCATGAGC

A1306		R: GTCGACAGTGGCAGGTGCATTACTA
	06-SlSNAT	F: GGTACCATGCAAACTCTCCACTTAGTATCCAC<br>R: GGATCCCTAATACATGGGGTACCAGAACATTCC
	06-SlAMST5	F: GAGCTCATGGCATTGCCTAATAATATTGGAGAC<br>R: GTCGACATCAAGGGTAAACCTCAATAACAGACC
	06-SlAMST7	F: GGTACCATGGCCAGTAACAACAATATTTGT<br>R: GGATCCTCTCTTTTGAAGCATAAGTAATCCTCA
	06-SlCOMT2	F: GAGCTCATGGAGAATGCAAATGGGGAAC<br>R: GTCGACCATGACTTGTGAAATTCAAGAATC
	RNAi(1)-SlPIF4	F: CGATGGTCTCACAACTGTGTTCAAGGAACATGTTGATGACG<br>R: CGATGGTCTCACAGGAAGCTGGCTCACCATTTGCC
	RNAi(2)-loop-SlPIF4	F: CGATGGTCTCACCTGCAGGTCTAGTTTTTCT<br>R: CGATGGTCTCAGCCCGGGCTCTGTAACTAT
	RNAi(3)-SlPIF4	F: CGATGGTCTCAGGGCAAGCTGGCTCACCATTTGCC<br>R: CGATGGTCTCATACATGTGTTCAAGGAACATGTTGATGACG
	RNAi(1)-SlphyB2	F: CAGTGGTCTCACAACATGCTGCATTTGAACAATCT<br>R: CGATGGTCTCACAGGGCATTTTCACTATAAGCAAT
	RNAi(2)-loop-SlphyB2	F: CGATGGTCTCACCTGCAGGTCTAGTTTTTCT<br>R: CGATGGTCTCAGCCCGGGCTCTGTAACTATC
	RNAi(3)-SlphyB2	F: CAGTGGTCTCAGGGCGCATTTTCACTATAAGCAAT<br>R: CAGTGGTCTCATACAATGCTGCATTTGAACAATCT
	RNAi(1)-SlSNAT	F: CAGTGGTCTCACAACGCTGCCGTTGTCAACCTTCA<br>R: CGATGGTCTCACAGGCTTGGAGATCATACACATCA
pBWA(V)HS	RNAi(2)-loop-SlSNAT	F: CGATGGTCTCACCTGCAGGTCTAGTTTTTCT<br>R: CGATGGTCTCAGCCCGGGCTCTGTAACTATC
	RNAi(3)-SlSNAT	F: CAGTGGTCTCAGGGCCTTGGAGATCATACACATCA<br>R: CAGTGGTCTCATACAGCTGCCGTTGTCAACCTTCA
	RNAi(1)-SlASMT7	F: CAGTGGTCTCACAACTATCACCAACAACAAGGAGA<br>R: CGATGGTCTCACAGGAATCAGAAATCAACAAATTA
	RNAi(2)-l	F: CGATGGTCTCACCTGCAGGTCTAGTTTTTCT

	oop-SlASMT7	R: CGATGGTCTCAGCCCGGGCTCTGTAACTATC
	RNAi(3)-SlASMT7	F: CAGTGGTCTCAGGGCAATCAGAAATCAACAAATTA<br>R: CAGTGGTCTCATACATATCACCAACAACAAGGAGA
	RNAi(1)-SlASMT5	F: CAGTGGTCTCACAACATGTTTTTGTACAATTTTCC<br>R: CGATGGTCTCACAGGTTGAGCAGCAAGGACTTCAT
	RNAi(2)-loop-SlASMT5	F: CGATGGTCTCACCTGCAGGTCTAGTTTTTCT<br>R: CGATGGTCTCAGCCCGGGCTCTGTAACTATC
	RNAi(3)-SlASMT5	F: CAGTGGTCTCAGGGCTTGAGCAGCAAGGACTTCAT<br>R: CAGTGGTCTCATACAATGTTTTTGTACAATTTTCC
	RNAi(1)-SlCOMT2	F: CGATGGTCTCACAACTGTGTTCAAGGAACATGTTGATGACG<br>R: CGATGGTCTCACAACTGTGTTCAAGGAACATGTTGATGACG
	RNAi(2)-loop-SlCOMT2	F: CGATGGTCTCACCTGCAGGTCTAGTTTTTCT<br>R: CGATGGTCTCAGCCCGGGCTCTGTAACTAT
	RNAi(3)-SlCOMT2	F: CGATGGTCTCAGGGCAAGCTGGCTCACCATTTGCC<br>R: CGATGGTCTCATACATGTGTTCAAGGAACATGTTGATGACG
ZMPL-DM-Cas9	Cas9-SlSNAT	F: CAGTGGTCTCATGCAATCTGTTCAACTGTTCCATC<br>R: CAGTGGTCTCAAAACTGCAACATCAGATCATGCAT
	Cas9-SlASMT7	F: CAGTGGTCTCATGCACAACGGGATGTTGGGCGAAA<br>R: CAGTGGTCTCAAAACGTGCCGTGGCGATGGCTATC
	Cas9-SlASMT5	F: CAGTGGTCTCATGCAACGAACGGGGCTAGACTCAA<br>R: CAGTGGTCTCAAAACCGTTCGCGAACCATTCACCG
	Cas9-proSlCOMT2	F: CAGTGGTCTCATGCATTACGTATGCTCACGAGCTA<br>R: CAGTGGTCTCAAAACGATGGTGCTGGAGCACGTAC
	pEAQ-8120	F: GTCGACATGGTTGTGCCAATTGTTGG<br>R: GAGCTCTTAAGGATAAACCTCAATTAGTGACCTTAATCC
	pEAQ-530	F: GTCGACATGGAAACAAATAATAATGTTGAGAGAGC<br>R: GAGCTCTTAAGGGAAAACTTGAATGAGGGAC

	pEAQ-510	F: GTATACATGGCCAATAAGAAGAACAACAT<br>R: GAGCTCTTTAAGGATAAACTTCCATTAGAGACCT
pEAQ-HT-DEST3	pEAQ-SlT5H-F	F: GTCGACATGGAAGCATCAATTCTACAGCTAC<br>R: GAGCTCTTACAACTTGTTGATGGTTGGAACAAG
	pEAQ-SlTDC280	F: GTCGACATGGGAACCCTTAATTCAAATAACAAC<br>R: GAGCTCCTAAAACACAATTTTTCTGAGCAAATCAT
	pEAQ-SlTDC860	F: GTCGACATGGGAAGCCTTGATTCCAA<br>R: GAGCTCTTAAAACACACTTTTTCTCAGCAAACC
	pEAQ-SlSNAT	F: GTCGACATGCAAACTCTCCACTTAGTATCCAC<br>R: GAGCTCCTAATACATGGGGTACCAGAACATTCC
	pEAQ-SlASMT7	F: GTCGACATGGCCAGTAACAACAATATTTGT<br>R: GAGCTCTCTCTTTTGAAGCATAAGTAATCCTCA
	pEAQ-SlASMT5	F: GTCGACATGGCATTGCCTAATAATATTGGAGAC<br>R: GAGCTCATCAAGGGTAAACCTCAATAACAGACC
	pEAQ-SlCOMT2	F: GTCGACATGGAGAATGCAAATGGGGAAC<br>R: GAGCTCCATGACTTGTGAAATTCAAGAATC

Table S4. Partial display of sequencing results of yeast screen library

ID	Note
Solyc06g074820.3	tonoplast intrinsic protein 1.1
Solyc06g036290.3	heat shock protein 90
Solyc05g052980.3	Protein phosphatase 2C (AHRD V3.3 *** Q8RVG0_TOBAC)
Solyc08g067030.3	transmembrane "protein," putative (Protein of unknown "function," DUF642) (AHRD V3.3 *** AT5G11420.1)
Solyc09g092380.3	S-adenosyl-l-homocysteine hydrolase
Solyc07g053830.3	ADP,ATP carrier "protein," mitochondrial (AHRD V3.3 *** ADT1_SOLTU)
Solyc02g085950.3	cell wall protein X77373
Solyc08g006890.3	Tubulin alpha chain (AHRD V3.3 *** TBA_PRUDU)
Solyc06g081980.1	Pyridoxal biosynthesis protein PDX1-like protein (AHRD V3.3 *** T2DNB9_PHAVU)
Solyc07g043580.4	bHLH transcription factor 052 (PIF4)
Solyc01g006430.3	Fatty acid desaturase (AHRD V3.3 *** M4QSE6_9ERIC)
Solyc04g049330.3	V-type proton ATPase subunit G (AHRD V3.3 *** A0A0B2RXB3_GLYSO)
Solyc06g059740.3	2 Alcohol dehydrogenase (AHRD V3.3 *** ADH_MALDO)
Solyc09g065590.3	auxin canalization protein (DUF828) (AHRD V3.3 *** AT3G22810.1)
Solyc09g010800.4	metallothionein II-like protein
Solyc06g071920.3	Glyceraldehyde-3-phosphate dehydrogenase (AHRD V3.3 *** C9DRQ8_SOLCH)
Solyc10g047410.1	Photosystem II CP43 reaction center protein (AHRD V3.3 *-* PSBC_SOLLC)
Solyc06g006080.3	thiamine biosynthesis protein ThiC

Solyc03g118780.3	Pathogenesis-related thaumatin family protein (AHRD V3.3 *** G7LDC8_MEDTR)
Solyc09g007850.3	RNA binding protein (AHRD V3.3 *** Q39209_ARATH)
Solyc10g048060.1	myosin XI D (AHRD V3.3 --* AT2G33240.5)
Solyc11g010230.2	Histone H3 (AHRD V3.3 *** K7VSQ3_MAIZE)
Solyc10g085880.1	Glycosyltransferase (AHRD V3.3 *** K4DF51_SOLLC)
Solyc09g097770.3	Glycine-rich protein (AHRD V3.3 *** D2K2T8_TOBAC)
Solyc08g067030.3	transmembrane "protein," putative (Protein of unknown "function," DUF642) (AHRD V3.3 *** AT5G11420.1)
Solyc09g092380.3	S-adenosyl-l-homocysteine hydrolase
Solyc07g043360.1	60S ribosomal protein L27 (AHRD V3.3 *** K4CEJ5_SOLLC)
Solyc01g087600.3	transmembrane "protein," putative (DUF288) (AHRD V3.3 *** AT2G41770.1)
Solyc02g082130.2	LOW QUALITY:Surfeit locus protein 6 (AHRD V3.3 *** AT5G05210.2)
Solyc12g035130.2	RNA helicase DEAD36
Solyc06g065230.3	N-acetyltransferase (AHRD V3.3 *** K7WTW4_SOLTU)
Solyc09g092380.3	S-adenosyl-l-homocysteine hydrolase
