# Supplementary material for: Understanding the mechanism of red light-induced melatonin biosynthesis facilitates the engineering of melatonin-enriched tomatoes
Source: Nat Commun. 2023 Sep 8;14:5525. doi: 10.1038/s41467-023-41307-5 (PMC10491657; doi:10.1038/s41467-023-41307-5)
Supplement: Supplementary file 3 — Description of Additional Supplementary Files [file 41467_2023_41307_MOESM3_ESM.pdf]

Description of Additional Supplementary Files

Supplementary Data 1 Transcription Factors from Yeast Library Screening
